# Role of Hybrid Deep Neural Networks (HDNNs), Computed Tomography, and Chest X-rays for the Detection of COVID-19

**DOI:** 10.3390/ijerph18063056

**Published:** 2021-03-16

**Authors:** Muhammad Irfan, Muhammad Aksam Iftikhar, Sana Yasin, Umar Draz, Tariq Ali, Shafiq Hussain, Sarah Bukhari, Abdullah Saeed Alwadie, Saifur Rahman, Adam Glowacz, Faisal Althobiani

**Affiliations:** 1 Electrical Engineering Department, College of Engineering, Najran University, Najran 61441, Saudi Arabia; miditta@nu.edu.sa (M.I.); asalwadie@nu.edu.sa (A.S.A.); srrahman@nu.edu.sa (S.R.); 2 Department of Computer Science, Lahore Campus, COMSATS University Islamabad, Lahore 54000, Pakistan; aksamiftikhar@cuilahore.edu.pk; 3 Department of Computer Science, University of OKara, Okara 56130, Pakistan; sanayaseen42@yahoo.com; 4 Department of Computer Science, University of Sahiwal, Sahiwal 57000, Pakistan; sheikhumar520@gmail.com (U.D.); Shafiq.hussain57@gmail.com (S.H.); 5 Computer Science Department, Sahiwal Campus, COMSATS University Islamabad, Sahiwal 57000, Pakistan; 6 Department of Computer Science, National Fertilizer Corporation Institute of Engineering and Technology, Multan 60000, Pakistan; Bukhari_sarah@nfciet.edu.pk; 7 Department of Automatic Control and Robotics, Faculty of Electrical Engineering, Automatics, Computer Science and Biomedical Engineering, AGH University of Science and Technology, 30-059 Kraków, Poland; adglow@agh.edu.pl; 8 Faculty of Maritime Studies, King Abdulaziz University, Jeddah 21577, Saudi Arabia; falthobiani@kau.edu.sa

**Keywords:** hybrid deep neural network (HDNNs), computed tomography (CT-scan), long short-term memory (LSTM), COVID-19

## Abstract

COVID-19 syndrome has extensively escalated worldwide with the induction of the year 2020 and has resulted in the illness of millions of people. COVID-19 patients bear an elevated risk once the symptoms deteriorate. Hence, early recognition of diseased patients can facilitate early intervention and avoid disease succession. This article intends to develop a hybrid deep neural networks (HDNNs), using computed tomography (CT) and X-ray imaging, to predict the risk of the onset of disease in patients suffering from COVID-19. To be precise, the subjects were classified into 3 categories namely normal, Pneumonia, and COVID-19. Initially, the CT and chest X-ray images, denoted as ‘hybrid images’ (with resolution 1080 × 1080) were collected from different sources, including GitHub, COVID-19 radiography database, Kaggle, COVID-19 image data collection, and Actual Med COVID-19 Chest X-ray Dataset, which are open source and publicly available data repositories. The 80% hybrid images were used to train the hybrid deep neural network model and the remaining 20% were used for the testing purpose. The capability and prediction accuracy of the HDNNs were calculated using the confusion matrix. The hybrid deep neural network showed a 99% classification accuracy on the test set data.

## 1. Introduction

The COVID-19 disease continues to have a shattering influence on the health and well-being of the global population, caused by infection in people with a critical respiratory condition. In this regard, the World Health Organization (WHO) declared an outbreak on 30 January 2020 as a “public health emergency of global concern” [1]. A critical phase in the fight against COVID-19 is the effective and optimal screening of the infected patients, so that these patients can receive instant care and treatment, as well as be quarantined to alleviate the spread of infection.

The leading COVID-19 detection and patient screening methods include antibody detection against the SARS-CoV-2 [2], reverse transcriptase-polymerase chain reaction (RT-PCR) [3] analysis, and artificial intelligence-based detection approaches [4]. These approaches are able to identify SARS-CoV-2 RNA from respirational samples collected by a variety of resources, such as oropharyngeal or nasopharyngeal modes. Although RT-PCR testing is an industry standard and an extremely precise test. It is, however, a very time-consuming, complex, and labor-intensive process that is limited in its application. Moreover, RT-PCR sensitivity of analysis is highly inconstant and is not stated in a perfect and reliable manner [5].

Real-time RT-PCR greatly improves the detection of SARS-CoV-2, due to its simple qualitative analysis and accuracy. However, this approach is used mostly in cases where the infection of diseases like (COVID) are needed to be detected in early stages, for early infection. It is also used in cases where RT-PCR is considered to be the main method for detecting COVID-19 and SARS-CoV, respectively. In addition to all these relevant facts, the important issue associated with real-time RT-PCR test is the risk of eliciting false-negative and positive results [5,6]. For example, it was observed that many ‘suspected’ cases with typical clinical characteristics of COVID-19 and identical specific CT images are not diagnosed [6,7,8,9,10,11]. Therefore, the negative results are intentionally excluded for the possibility of a COVID-19 infection and it should not be used as the only criterion that is considered for treatment and patient management decisions. Consequently, it is reported that the combination of real-time PCR with clinical features, which could possibly help manage SARS-CoV-2 and COVID-19 outbreak. Moreover, there are many factors highlighted for these reasons that are mentioned in [12,13,14,15].

Recently, neural networks gained excessive achievements in the field of medical imaging, due to their self-learning capabilities and high aptitude for automatic feature extraction [16]. Especially, deep neural networks can distinguish infectious and virus-related pneumonia for chest radiographs [17,18,19,20,21]. Therefore, in this article, we introduce a hybrid deep neural network (HDDNs) for the diagnosis of COVID-19, using CT and X-ray images. This network classifies CT images for healthy and COVID patients, and determines the infection possibility of COVID-19. These outcomes might significantly contribute to the primary screening of COVID-19 patients.

There are numerous benefits to leveraging computed tomography images for COVID-19 screening for the universal COVID-19 epidemic. These benefits are even more relevant, specifically in remote and highly affected areas and are discussed as follows. (1) Fast triaging—computed tomography imaging facilitates fast triaging of patients doubted of COVID-19, and can be accomplished in parallel epidemiological testing, which is time consuming to provide assistance to great volumes of patients in highly affected areas. Moreover, computed tomography imaging can be quite efficient for triaging in geographical regions where patients are educated to stay home until the inception of radical symptoms. Meanwhile, anomalies are frequently seen at the time of demonstration when patients suspect that the COVID-19 reached clinical sites. (2) Accessibility and user-friendly—computed tomography imaging is available in many clinical sites and imaging centers, as it is an ordinary imaging tool in most healthcare structures and is much more readily accessible in developed countries as a cost-effective product. (3) Flexibility—the presence of flexible CT scan systems means that imaging can be executed within a quarantine room, which in turn decreases the risk of COVID-19 spread.

The fast spread and late diagnosis of COVID-19 stunned the world and influenced the lives of billions of people, from both a safety and an economic perspective. Existing testing kits are limited in number and can test only a few patients. Additionally, usage of fake testing kits in medical industry is also quite common, which not only results in waste of money but also incorrect test results. Hence, designing an automated diagnosis system is essential for providing an efficient and reliable solution. The proposed hybrid technique provides an automated detection for COVID-19 patients that can save billions of people’s lives and medical professional’s valuable time, which they invest for examining chest X-rays, to form an opinion.

The major contributions of this study that makes it unique over traditional machine-learning or deep-learning techniques are given below.

1. State-of-the-art hybrid COVID-19 detection by using a multi-model and multi-data approach [22,23,24]. Including multi model and multi data has its own cost, as we need more data and complex models for performing the classification task. However, they add to the efficacy of the model, as the model can exploit more rich information for the classification task. Particularly, the data from different modalities complement each other. Therefore, it can be said that this is a general phenomenon, which is also evident in many earlier studies, involving multi-model/multi-data studies [25,26].
2. Multimodal dataset (CT and X-rays images), which provides more accurate and reliable results in comparison to the single CT image data set or single X-ray datasets.
3. The hybrid deep neural network model is a mixture of two deep-learning models (LSTM + CNN) and is capable of accurately classifying COVID Patients. The proposed CNN- and LSTM-based layer arrangements show a noteworthy performance, as compared to previous deep neural network architectures, by automatically learning the patterns in the COVID-19 data, which is fruitful for the classification of COVID patients from healthy controls.
4. The automatic feature extraction mechanism better learns the features compared to previous COVID studies.
5. To the best of our knowledge, it is the first COVID-19 detection technique that simultaneously works on the multi-model and multi-data approach and gives higher accuracy in comparison to the existing COVID-19 detection techniques.

The performance comparison of the proposed HDDNs with existing COVID-19 detection techniques is shown in Table 1.

The rest of the paper is organized as follows. Section 2 describes the methodology, which is further subdivided into experimental data acquisition, preprocessing, and the hybrid deep neural network (HDNNs) architecture. Section 3 describes the experimental results of the proposed technique that further elaborates the quantitative, qualitative analysis, and comparison of the proposed HDNNs model, with the existing techniques. In the end, the conclusion section reveals the potential of the hybrid deep neural network (HDNNs), by concluding the different performance parameters and accuracy comparison.

## 2. Methodology and Deliverables

### 2.1. Experimental Data Acquisition

In this study, the chest X-ray images dataset that we refer to as “Hybrid-COVID” with dimensions of (1080 × 1080 pixels) was used to test and train our hybrid deep neural network architecture (HDNNs). This was designed by extracting the COVID-19 data from five different sources—GitHub [41], COVID-19 radiography database [42], Kaggle [43], COVID-19 image data collection [44], and Actual Med COVID-19 Chest X-ray Dataset [45], which are open-source and publicly available data repositories.

Before further usage, we combined all 5 datasets into a single dataset that consisted of 5000 patients (57% male, 32% female), containing 3500 infected and 1500 healthy controls, with an age group of 38–55 years. The selection of these five databases to create “Hybrid-COVID” was directed by the fact that all these five databases are open source and fully available to the clinicians, research community, and general public, and fulfills the diagnostic criteria of COVID-19, defined by the World Health Organization (WHO).

### 2.2. Preprocessing

Noise always exists in digital images and it is most challenging to remove noise without previous knowledge of filtering techniques. The acquired COVID-19 data were polluted with different types of noise. This noise occurred due to many reasons, including monitoring devices, patient movement, and device error. It is necessary to sanitize these data from noise because it affects the classification accuracy of the model. To remove these lower quality data from actual images, we analyzed the results of several researchers. An iterative mean filter for image denoising was used in [46], which was based on the LMS (least mean squares) algorithm and decreased the noise in digital image processing. Rai et al. [47] endorsed the use of a hybrid adaptive algorithm, based on wavelet transform and independent component analysis for denoising of MRI images, and for efficient suppression of the interference in images. A general model for noise contamination can be described by Equation (1).

P (*n*) = Q (*n*) + T (r) (1)

where P (*n*) and Q (*n*) are samples of the COVID data, including and excluding noise, respectively; r represents the source inference, and T is an unknown transfer function.

The Kalman filter [9] is an efficient recursive data processing algorithm, which was extensively used in many applications, such as industrial control systems, radar tracking, aero-engine analysis, and intellectual robots. Kalman filter works well in reducing noise, while preserving the underlying structure of an image, when compared to the other said filter. We used it in our study because the Kalman filtering method recursively uses past data and gives more accurate results than a filtering method based only on incoming measurements. In comparison to [48,49], which used deep-learning approach to denoise the CT images and is well-developed for medical image denoising, we used the Kalman filter in our article due to the multi-imaging data, recursive data processing, prior predicted value observation, and polluted region detection, which gives acceptable performance using the Kalman filter. In the Kalman filter derivation formula, joins an Adaptive Predictor Filter (APF) and Discrete Wavelet Transformation (DWT) to identify pure noise. Moreover, our other approaches like [50,51,52,53] also support our words. 

The noise removal model proposed in the present study included the following steps—(1) image decomposition, (2) noise detection (RA) region detection, (3) polluted parts prediction, and (4) image renovation. The DWT was used to decompose the images and identify the regions. The DWT decomposition finds the low-frequency parts and nonstationary time-series, which are then divided into several approximate stationary time-series. The actual image is predicted by the decomposed images, by applying the conventional Kalman filter. The adaptive filter is applied to improve prediction and to estimate future values based on the previous one.

We used the following Kalman discrete-time model to remove the noise with the state Equation (2).

(2) $$P_{k+1}\;=\;Q_{k\;\;}p_{k\;}\;+s_{k}$$

and the analysis equation

(3) $$m_{k}=R_{k}P_{k}+u_{k}$$

where $p_{k+1}$ is the state variable, $m_{k}$ is the analysis variable, $Q_{k\;}$ and $R_{k}$ are matrices with n number of rows and m number of columns, $s_{k}$ is the modeling error noise and $u_{k}$ is the analysis error noise, respectively. 

We only focus on the $s_{k}$, which is Gaussian noise, and $u_{k}$, which is a non-Gaussian noise.

By considering Equations (2) and (3), the Kalman filtering model supposes that $s_{k}$ and $u_{k}$ are both Gaussians, with the following covariance matrix:

(4) $$E\;⟦s_{i\;sk^{T}}⟧=\{\begin{array}{c} S_{k\;\;}\;\;for\;\;\;\;\;\;i=k\\ 0,\;\;\;\;for\;\;\;\;\;\;\;i\;\neq k \end{array}$$

(5) $$E\;⟦u_{i\;uk^{T}}⟧=\{\begin{array}{c} T_{k\;\;}\;\;for\;\;\;\;\;\;i=k\\ 0,\;\;\;\;for\;\;\;\;\;\;\;i\;\neq k \end{array}$$

Let −$p_{k}$ be the priority estimation, which is the approximation of $p_{k}$ from m_0_, m_1_, ⋯, mk−1, and let $p_{k}$ be the posterior estimation, which is the approximation of $p_{k}$ from m0, m1, ⋯, mk.

(6) $$E\{-p_{k\;}\}\;=\;E\{p_{k}\}$$

and where E signifies expectation and $Q_{k}$ is from (2)

(7) $$-p_{k\;}+1\;=\;Q_{k\;\;}p_{\underset{o}{k}}$$

The Kalman filter supposes that the posterior estimation is expressed as the prior estimate corrected by the measurement data:

(8) $$p_{\underset{o}{k}}\;=\;-p_{k\;}+\;\mathrm{Kk}\;(m_{k\;}\;-\;R_{k\;}p_{\underset{o}{k}})$$

for n rows and m columns matrix Kk represents the Kalman gain.

Note that $R_{k}$ is from (3). 

The Kalman gain Kk is resolved by decreasing E ($p_{ko\;}$ − $p_{k}$)2 

Note that

(9) $$E\;(p_{\underset{o}{k}}-p_{k\;})\;T\;(p_{\underset{o}{k}}\;-p_{k\;})\;=\;\mathrm{Tr}\{\mathrm{Zk}\}$$

where Tr signifies the trace operator and the n cross n covariance matrix Z_k_ is presented as follows

(10) $$-Z_{k\;}\;=\;E\;(p_{k\;}-(-p_{k\;}))(\;p_{k\;}\;-\;(-p_{k\;})T)$$

Putting (9) into (10), we get

(11) $$Z_{k\;}\;=\;(I\;-\;K_{k\;}R_{k\;})\;-Z_{k\;}\;(I\;-\;K_{k\;}R_{k\;})\;T\;+\;(K_{k\;}R_{k\;}K_{k\;})T$$

By (10) and (12), $Z_{k}$ can be simplified as

(12) $$Z_{k\;}=\;(I\;-\;K_{k\;}R_{k})\;-Z_{k\;}$$

Combining (9), (10), and (11), thus, we filter the image from the conventional Kalman filter.

Finally, noise that had a high effect on the chest X-ray-based COVID data, was removed from the raw images, and the data were ready for further processing.

### 2.3. Proposed Hybrid Deep Neural Network Architecture (HDNNs)

In this study, a hybrid deep neural network architecture based on a convolutional neural network (CNN) and LSTM (Long short-term memory) for COVID detection is proposed. These two models are deep learning-based models, which is a sub-field of artificial intelligence. The two deep learning models are well-suited to classifying, processing, and making predictions, which resulted in extraordinary performance in automatic feature extraction from images datasets [23,26]. We picked the CNN model, because of their automated feature learning, and used LSTM to deal with the vanishing gradient problem, which occurs when training neural networks. The proposed HDNN architecture is based on the three distinctive arrangements of diverse layers (convolutional layers, pooing layers, and dropout layers), and one LSTM layer that was assessed on the COVID-19 datasets used in this article. The convolutional layer was the major building block of CNN and was used to filter out the discriminating features from the original images. The pooling layer was utilized to reduce the dimensionality of the data by using the sliding window approach, which is based on the size of the window. The dropout layer was used to prevent a model from over-fitting.

The proposed layer arrangements showed a noteworthy performance, as compared to the previous deep neural network architectures, by automatically learning the patterns in COVID-19 data that is fruitful for the classification of COVID patients from healthy controls.

The mathematical representation of the proposed model and their layer combination is stated below.

Convolutional Layer:

(13) $$C\left(p,q\right)=\left(w*z\right)\left(p,q\right)={\sum^{\;}}_{i}\;{\sum^{\;}}_{j}\;z\left(i,j\right)w\left(p-i\right),\left(q-j\right)$$

where *w* = image, *z* = kernel, *p* and *q* are the indices of rows and columns of the resultant matrix.

Equation (14) describes the mathematical functionality of how the feature detector shifts according to the input.

Convolution function:

(14) $$\left(w*z\right)\left(t\right)=\int_{-\propto }^{\propto }w\left(⊺\right)z\left(⊺-z\right)d_{t}={\left(w*z\right)}^{\Delta }\int_{-\propto }^{\propto }f\left(t-⊺\right)z\left(z\right)d_{t}$$

where *t* is the time index and is an integer, *w* and *z* are integers.

A common engineering convention is:

(15) $$w\left(t\right)*z{\left(t\right)}^{\Delta \;}\int_{-\propto }^{\propto }w\left(t-⊺\right)g\left(t-⊺\right)d_{t}$$

This research was implemented in Python by applying a hybrid deep neural network, referred to as (HDNNs), at the X-ray and computed tomography (CT) images. CT is a non-invasive imaging approach that has a capability to capture specific conditions in the lungs that are associated with COVID-19. Thus, we analyzed it by the most appropriate deep neural network approach, which is an effective tool for the primary analysis of COVID-19. Artificial intelligence using deep neural networks already attained greater performance in the field of radiology [10]. Past research effectively applied survey-based and transcriptase-polymerase chain reaction methods, to identify pneumonia in pediatric chest radiographs, to distinguish pathological and bacteriological pneumonia in 2D pediatric chest radiographs [11].

**Figure 1:**
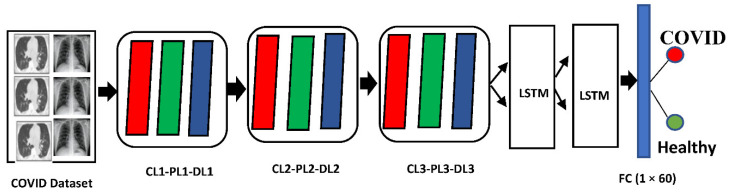
Hybrid Deep Neural network (HDNNs) architecture for COVID-19 detection consists of a dropout layer (DL), a convolutional layer (CL), a pooling layer (PL) with LSTM blocks, and a fully connected (FC) layer.

**Figure 2:**
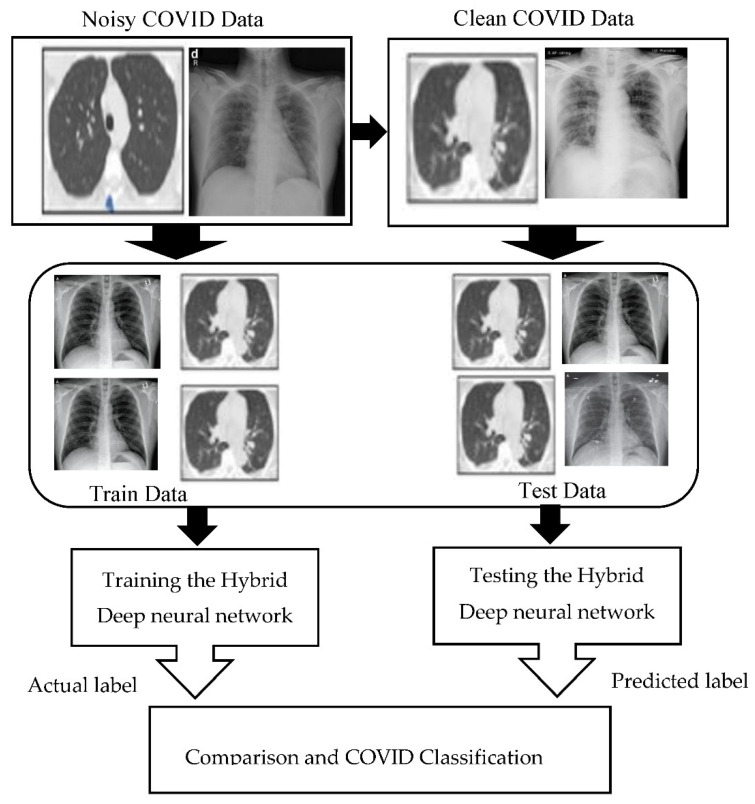
The block level representation of our proposed technique by using hybrid deep neural network (HDNNs) and chest X-ray.

In this article, HDNNs is applied to computed tomography (CT) [12], which achieved a higher classification accuracy than the other existing techniques in the literature. The framework of HDNNs is shown in Figure 1. This framework was trained by using the transfer learning approach that automatically extended from previous training and then reused it in further diagnosis. The infection probability of COVID-19 was formulated using two major Python libraries Keras and Tensor Flow. Ultimately, the chest, CT, and HDNNs provide a consistent and fast methodology for the identification of COVID-19 patients. The block-level representation of our proposed technique by using the hybrid deep neural network (HDNNs) and chest X-ray is shown in Figure 2.

The proposed hybrid deep neural network divides the COVID data with the 1-s window size and 256 samples, by taking the data as a time-series format. As the sampling rate was 256 samples per second, every COVID fragment enclosed 256 data points (window length). Empirical evaluation was done for the selection of window size and it was observed that a window size of 1 s gave significant results. The input data dimension of COVID datasets is set to 256 × 64 for every instance of class. Furthermore, the input COVID data are segmented into a training and testing set, with a ratio of 80 and 20 percent, respectively. Initially, the training dataset was passed to the hybrid deep neural network models for the classification of COVID and healthy subjects, and then the testing datasets was applied to evaluate the classifier performance, using several performance metrics like accuracy, precision, recall, and F1-score.

#### Evaluation Criteria

The four different metrics were used to evaluate the proposed method. These metrics were accuracy, precision, recall, and F1 score.

The mathematical representation of the performance metrics is shown below.

Accuracy = (tn + tp)/(tp + fn + fp + tn)(16)

Precision = tp/(tp + fp)(17)

Recall = tp/(tp + fn)(18)

F1 = 2 × Precision × recall/precision + recall(19)

where “RN” refers to true positive, “tn” shows true negative, “fp” represents false positive, and “fn” represents false negative.

### 2.4. Potential Risk Imperial to the Development of Progress & Related Risk Strategy

The potential risks that we faced during development were finding a balance between sensitivity and specificity, which was an incredible challenge, because infective diseases like COVID-19 transfer quickly.

**Figure 3:**
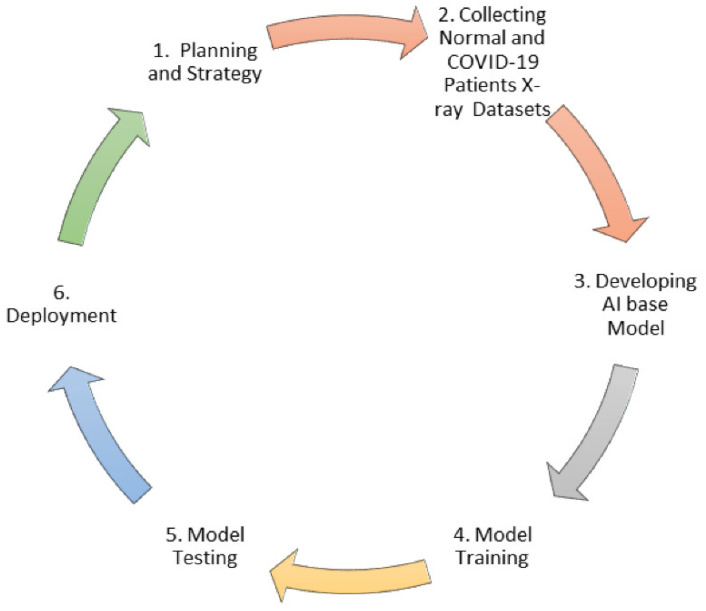
Implementation flow of data collection and deliverable.

The implementation flow of data collection and deliverable is represented in Figure 3.

## 3. Experimental Results

To estimate the effectiveness of the proposed HDNNs, we executed both quantitative and qualitative analysis, to develop a good understanding of its identification and decision-making behavior.

### 3.1. Quantitative Analysis

To examine the proposed HDDNs performance in a quantitative manner, we calculated the test accuracy, as well as a positive predictive value (PPV) and sensitivity for each type of contamination, on the above-mentioned COVID-19 X-ray dataset. The test sensitivity and positive predictive value (PPV) ratio for normal, non-COVID (Pneumonia), and COVID patients, along with the applied architecture, are shown in Table 2 and Table 3, respectively. The results showed that HDDNs attained a good test accuracy (99%) for detecting COVID-19 patients, consequently emphasizing the effectiveness of leveraging a human-machine cooperative design scheme for making highly-customized deep neural network architectures. The performance of the proposed HDDNs model for COVID-19 detection was also evaluated with the help of a confusion matrix, which is often used to evaluate the accuracy of machine-learning classifiers. It consists of a set of rows and tables in which each row of the confusion matrix shows the number of instances in the predicted class, while the columns represent the number of instances in an actual class or vice-versa.

### 3.2. Qualitative Analysis

**Figure 4:**
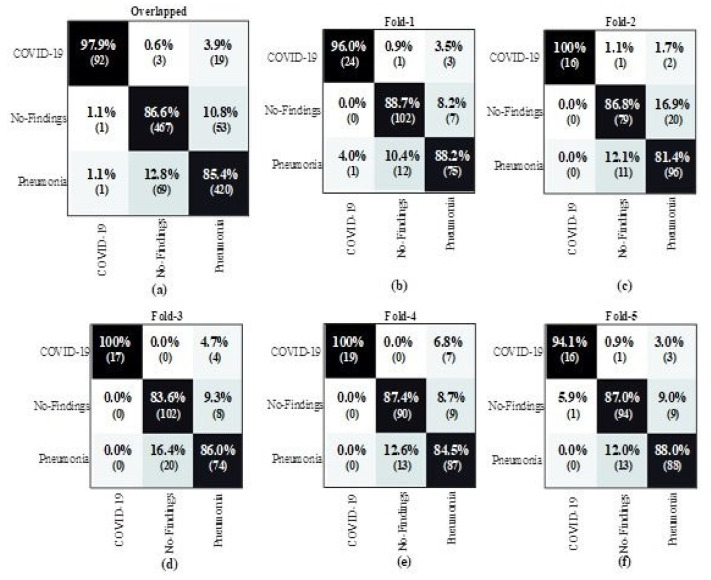
The 5-fold confusion matrix results of the multi-class classification task. (**a**) Overlapped Confusion Matrix, (**b**) 1-Fold Confusion Matrix (CM), (**c**) 2-Fold Confusion Matrix (CM), (**d**) 3-Fold Confusion Matrix (CM), (**e**) 4-Fold Confusion Matrix (CM), and (**f**) 5-Fold Confusion Matrix (CM).

This section presents the detailed data distribution used for the proposed HDDNs framework, to get a better understanding of how HDDNs make decisions. It authenticates whether it is making recognition decisions, based on significant information (data) or on inaccurate information, i.e., biased decisions based on inappropriate data. Such situations are very problematic and difficult to track. A dataset of over 5000 COVID patients was used in this study. The data distribution was analyzed to train and test the X-ray and CT images. The distribution of the X-ray images for COVID-19 detection is shown in the first half of Table 4. Similarly, the distribution of CT images for COVID-19 detection is shown in the second half of Table 4. The training and testing images for all 3 categories (normal, pneumonia, and COVID-19) are shown separately. It can be seen from the table that almost 80% of the data was used for training and almost 20% of the data was used for testing. The evaluation of the training performance of the hybrid deep neural network for the COVID-19 dataset was also conducted by importing the python library Keras, and training loss and accuracy of the COVID-19 dataset was also measured for tracking the training performance. The resultant output of the proposed method is presented in the form of a confusion matrix in Figure 4.

### 3.3. Comparison of HDNNs with the Existing COVID-19 Detection Techniques

**Figure 5:**
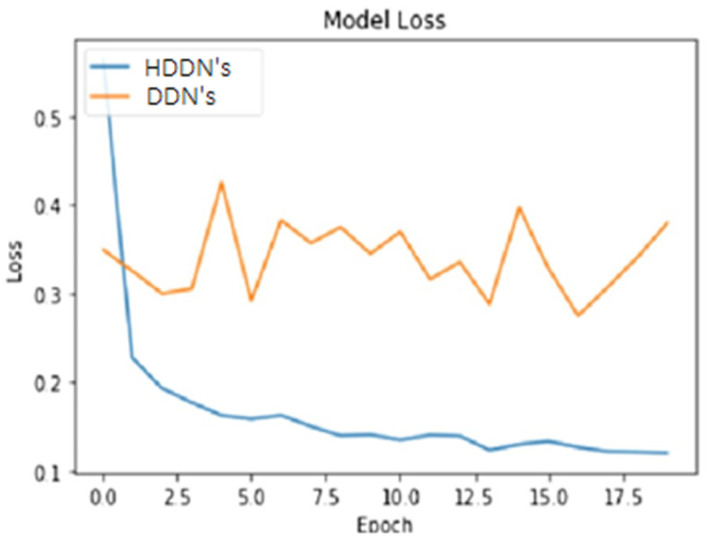
Loss of COVID-19 dataset against the number of COVID-19 CT and chest X-ray samples for the deep neural network (DNNs) and hybrid deep neural network (HDDNs).

**Figure 6:**
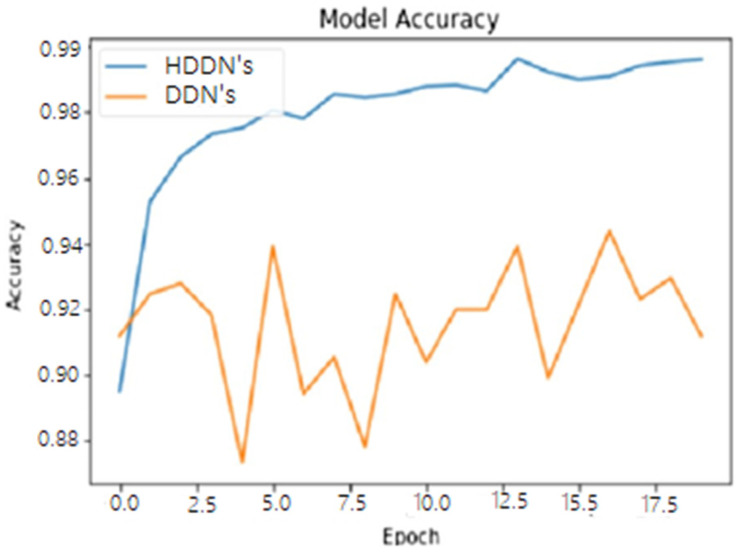
Accuracy of COVID-19 dataset against the number of COVID-19 CT and chest X-ray samples for the deep neural network (DNNs) and hybrid deep neural network (HDDNs).

To make a comparison of our state of the art HDDN’s approach with the existing COVID-19 detection techniques and to prove the originality of our work, we selected the deep neural network (DNN’s) approach as the benchmark. First, we evaluated both techniques at the raw CT and X-ray images to calculate the loss that depicts the inaccuracy of the model result, and wrongly classifies the presence of disease that does not exist in reality. The graphical behavior of DNN and HDNNs is shown in Figure 5, against the number of COVID-19 CT and chest X-ray samples. The classification accuracy of both the DNN and HDNNs neural network models is analyzed in Figure 6. The hybrid neural network (HDNNs) model with long short-term memory (LSTM) led the DNN to have a 99% classification accuracy.

## 4. Conclusions

This article revealed the potential of a hybrid deep neural network (HDNNs) for the automatic diagnosis of COVID at computed tomography and chest X-ray data. The benefit of the proposed HDNNs over the traditional deep learning and machine learning frameworks is the use of multi model and multi data. After performing the analysis at the COVID-19 X-ray datasets by using the hybrid deep neural network and computer tomography (CT), it was concluded that the hybrid deep neural network could accurately identify COVID-19 and discriminate it from patients with pneumonia. It showed excellent sensitivity for identification of COVID-19. In comparison to previous techniques used for COVID detection, our proposed model HDDNs had a 99% classification accuracy. In future, we believe that it will prove to be an essential tool for COVID-19 identification in endemic areas.

## Figures and Tables

**Table 1:** Performance comparison of existing COVID-19 detection techniques with HDDNs, in which the shaded area represents the chest X-rays-based techniques that are used as a benchmark for this study.

Authors	Published	Technique Summary	Performance
**Xiao, L., et al.** [27]	31 July 2020	Artificial intelligence-assisted tool using computed tomography (CT) imaging to predict disease severity.	Accuracy: 81.9%
**Li et al.** [28]	19 March 2020	Artificial intelligence approach with chest X-ray	Per-scan sensitivity and specificity: 87% and 92%
**Dansana, D. et al.** [29]	28 August 2020	CNN based methods using CT and X-ray images	Validation accuracy: (91%)
**Chen, J., et al.** [30]	1 March 2020	Deep Learning and CT images based method for COVID detection	Accuracy: 95.24%,
**Zhang et al.** [31]	28 June 2020	Deep learning with chest X-ray	Accuracy: 83.61% and sensitivity: 71.70%
**Zhang, K., et al.** [32]	3 September 2020	AI system to diagnose COVID-19 pneumonia using CT scans	Accuracy: 80%
**Narin, et al.** [33]	12 July 2020	deep CNN using X-ray images	Accuracy: 98%
**Acar, E., et al.** [34]	14 June 2020	Deep learning-based models for detecting COVID-19 from computed tomography (CT) images	Accuracy: 98.8%
**Ozturk et al.** [35]	18 June 2020	Deep Neural network with X-ray images	Accuracy: 98.08% and 87.02% for binary and multi-classes, respectively
**Soares, L., et al.** [36]	2 July 2020	Automatic Detection of COVID-19 Cases on X-ray images Using Convolutional Neural Networks	Accuracy 81%
**Goel, C., et al.** [37]	17 August 2020	Deep Network Architecture for COVID-19 Detection Using Computed Tomography Images	Accuracy 96.78%
**Afshar, P., et al.** [38]	28 September 2020	COVID-19 Computed Tomography (CT) Scan using Machine Learning and Deep Learning	Accuracy 91%
**Song, Y., et al.** [39]	25 February 2020	Deep learning-based CT diagnosis system	Accuracy: 0.99 and sensitivity: 0.96
**Shah, V., et al.** [40]	11 July 2020.	Diagnosis of COVID-19 using CT scan images and deep learning techniques	Accuracy: 94.52%
**Our Study**	10 January 2021	Hybrid Deep Neural Networks (HDNNs), CT images and Chest X-rays for the detection of COVID-19	Classification accuracy: 99%

**Table 2:** Sensitivity for Normal, Pneumonia Patient, and COVID Patient.

Sensitivity			
Neural Network Architecture	No Findings	Pneumonia Patient	COVID-19 Patient
Recurrent Neural Networks (RNN)	78%	80.5%	81.4%
Deep Belief Networks (DBNs)	82.3%	84%	83.0
Deep Neural Network (DNNs)	81.5%	86.7%	87%
Hybrid Deep Neural Network (HDNNs)	88.1%	99.5%	99%

**Table 3:** Positive predictive value (PPV) for each infection type.

Positive Predictive Value (PPV)			
Neural Network Architecture	No Findings	Pneumonia Patient	COVID-19 Patient
Recurrent Neural Networks (RNN)	68.1%	70.5%	51.4%
Deep Belief Networks (DBNs)	72.3%	74%	75.0
Deep Neural Network (DNNs)	81%	84.7%	86%
Hybrid Deep Neural Network (HDNNs)	89.%	96.5%	98.7%

**Table 4:** Distribution of X-ray and CT images for different contamination types.

Subject Type	Number of Images (X-ray)	
	**Training**	**Testing**
Normal	300	200
Pneumonia	800	200
COVID-19	1000	200
	**Number of Images (CT)**	
Normal	400	200
Pneumonia	500	200
COVID-19	800	200

## Data Availability

This study does not report any data which required external approval.
